# Trans-ethnic follow-up of breast cancer GWAS hits using the preferential linkage disequilibrium approach

**DOI:** 10.18632/oncotarget.13075

**Published:** 2016-11-04

**Authors:** Qianqian Zhu, Lori Shepherd, Kathryn L. Lunetta, Song Yao, Qian Liu, Qiang Hu, Stephen A. Haddad, Lara Sucheston-Campbell, Jeannette T. Bensen, Elisa V. Bandera, Lynn Rosenberg, Song Liu, Christopher A. Haiman, Andrew F. Olshan, Julie R. Palmer, Christine B. Ambrosone

**Affiliations:** ^1^ Department of Biostatistics and Bioinformatics, Roswell Park Cancer Institute, Buffalo, NY, USA; ^2^ Department of Biostatistics, Boston University School of Public Health, Boston, MA, USA; ^3^ Department of Cancer Prevention and Control, Roswell Park Cancer Institute, Buffalo, NY, USA; ^4^ Slone Epidemiology Center, Boston University, Boston, MA, USA; ^5^ Department of Epidemiology, Gillings School of Global Public Health, University of North Carolina at Chapel Hill, Chapel Hill, NC, USA; ^6^ Cancer Prevention and Control, Rutgers Cancer Institute of New Jersey, New Brunswick, NJ, USA; ^7^ Department of Preventive Medicine, Keck School of Medicine, University of Southern California/Norris Comprehensive Cancer Center, Los Angeles, CA, USA

**Keywords:** causal variant, genome-wide association studies, fine-mapping

## Abstract

Leveraging population-distinct linkage equilibrium (LD) patterns, trans-ethnic follow-up of variants discovered from genome-wide association studies (GWAS) has proved to be useful in facilitating the identification of *bona fide* causal variants. We previously developed the preferential LD approach, a novel method that successfully identified causal variants driving the GWAS signals within European-descent populations even when the causal variants were only weakly linked with the GWAS-discovered variants. To evaluate the performance of our approach in a trans-ethnic setting, we applied it to follow up breast cancer GWAS hits identified mostly from populations of European ancestry in African Americans (AA). We evaluated 74 breast cancer GWAS variants in 8,315 AA women from the African American Breast Cancer Epidemiology and Risk (AMBER) consortium. Only 27% of them were associated with breast cancer risk at significance level α=0.05, suggesting race-specificity of the identified breast cancer risk loci. We followed up on those replicated GWAS hits in the AMBER consortium utilizing the preferential LD approach, to search for causal variants or better breast cancer markers from the 1000 Genomes variant catalog. Our approach identified stronger breast cancer markers for 80% of the GWAS hits with at least nominal breast cancer association, and in 81% of these cases, the marker identified was among the top 10 of all 1000 Genomes variants in the corresponding locus. The results support trans-ethnic application of the preferential LD approach in search for candidate causal variants, and may have implications for future genetic research of breast cancer in AA women.

## INTRODUCTION

Genome-wide association studies (GWAS) premised on the “common disease, common variants” hypothesis have made great strides in identifying common genetic variants associated with a variety of phenotypes [1]. Typically, the GWAS-identified variants for any particular phenotype cumulatively explain only a small portion of the phenotypic variation [2]. One explanation for the so-called missing heritability problem is that the variants identified by GWAS are often only proxies of the causal variants that still remain to be discovered [3, 4]. The association signal attenuates as the linkage disequilibrium (LD) between the causal variant(s) and the GWAS-discovered variant decreases, particularly when the causal variant(s) are rare in the population. Until recently, the majority of GWAS have focused on populations of European descent, where the causal variants can be far from the associated marker due to strong LD, making it difficult to localize the causal variants [5, 6]. Thus, trans-ethnic follow-up studies in populations with lower LD are becoming more common [7–13]. Studies in populations with lower average LD often yield shorter distances between causal variants and the associated marker, helping to narrow down the causal variants underlying the disease associations [5, 14].

We previously developed the novel preferential LD approach to identify causal variants that drive the GWAS signal of interest from a comprehensive genome-wide variant catalog [15]. This approach is premised on the notion that the LD between the causal variant(s) and the GWAS-discovered variant is stronger than the LD between the causal variant(s) and most other variants interrogated in the GWAS, even if the causal variants are rare and only weakly linked to the GWAS-discovered variant. The increasing number of publications where causal variants are in only weak LD with the GWAS hits emphasizes the need for approaches beyond the absolute magnitude of the LD [16–29]. Our approach selects candidate causal variants at the locus of the GWAS-discovered variant from the variant catalog and prioritizes them based on the tagging specificity of the candidate variants by the GWAS-discovered variant, as well as functional importance of the candidate variants. We showed that the approach could successfully pinpoint the known causal variants of diverse traits when the discovery population and follow-up population were from the same ethnic group [15]. We anticipated that the preferential LD approach would also perform well in a trans-ethnic setting, which leverages the benefit of shorter LD structure. As the preferential LD approach does not rely on any phenotypic information, another advantage of the approach is its ability to follow up GWAS of various phenotypes using the same comprehensive variant catalog, such as the 1000 Genomes project [30, 31] or other large-scale sequencing efforts. This feature allows the approach to make the most use of the rapidly accumulating variant data from next-generation sequencing.

In this study, we evaluated the performance of the preferential LD approach in an African American (AA) population, following up breast cancer GWAS hits. We carried out the study in the African American Breast Cancer Epidemiology and Risk (AMBER) consortium, which provides rich genetic and epidemiological resources for investigating breast cancer risk in AA women [32–36]. To date, a large number of GWAS and ensuing meta-analyses have been performed on breast cancer susceptibility, with more than 90 loci meeting the stringent criteria of genome-wide significance level identified [37–39]. However these GWAS were predominantly performed in European populations, with only two conducted among individuals of African ancestry [40, 41]. Even in the well-studied European populations, collectively, the GWAS-discovered variants only account for an estimated 16% of breast cancer heritability [39]. The large missing heritability emphasizes the need to pinpoint causal variants or better markers of breast cancer.

We first sought to evaluate the 74 GWAS-identified breast cancer risk variants (Supplementary Table S1) in 8,315 AA women (3,648 cases, including 1,977 ER+ cases and 1,092 ER- cases, and 4,667 controls) from the AMBER consortium [34–36]. We then followed up those GWAS signals that were replicated in AAs by utilizing the preferential LD approach and the comprehensive variant catalog of African population from 1000 Genomes Project, to search for candidate causal variants or better breast cancer markers in an AA population. The variants selected by the preferential LD approach were genotyped in the AMBER consortium and then compared with all neighboring 1000 Genomes variants in evaluation of the approach's performance in a trans-ethnic setting.

## RESULTS

### Replication of GWAS-discovered variants in AA women from the AMBER consortium

Out of the 74 GWAS-discovered variants, only 18 were associated with overall breast cancer risk in AMBER with nominal p-value < 0.05 (Table 1). All of them had association directions consistent with previous reports. The replication rate was higher for the variants discovered initially in populations of African ancestry [53] (4/7 or 57.1%) than those discovered in populations of non-African ancestry (14/67 or 20.9%).

**Table 1:** Replicated GWAS-discovered variants in AMBER imputation data

GWAS-discovered Variants	Region	Neighboring Genes	GWAS Population	Risk allele	RAF^a^	Imputation Quality^b^	OR	p-value^c^
Overall breast cancer risk								
rs4849887	2q14.2	LOC84931, GLI2	European ancestry	C	0.7124	0.9967	1.1086	6.85E-03
rs13000023	2q35	TNP1, DIRC3	African American	G	0.8480	1.0079	1.1787	6.13E-04
rs16857609	2q35	DIRC3	European ancestry	T	0.2483	1.0099	1.1196	4.16E-03
rs13387042	2q35	TNP1, DIRC3	European ancestry	A	0.7291	1.0121	1.0841	0.0365
rs10069690	5p15.33	TERT	European ancestry	T	0.5955	1.0237	1.1107	2.40E-03
rs1432679	5q33.3	EBF1	European ancestry	C	0.7989	1.0163	1.1373	2.58E-03
rs9693444	8p12	DUSP4, LINC00589	European ancestry	A	0.3833	0.9835	1.0781	0.0339
rs1011970	9p21.3	CDKN2B-AS1	European ancestry	T	0.3312	1.0244	1.0740	0.0473
rs2981578	10q26	FGFR2	African American	C	0.8542	1.001	1.2520	4.99E-06
rs2981579	10q26.13	FGFR2	European ancestry	A	0.6069	1.0172	1.1187	1.30E-03
rs1219648	10q26.13	FGFR2	European ancestry	G	0.4286	1.0068	1.0766	0.0324
rs2981582	10q26.13	FGFR2	European ancestry	A	0.4802	1.0005	1.0716	0.0438
rs3817198	11p15.5	LSP1	European ancestry	C	0.1651	0.9933	1.0962	0.0472
rs609275	11q13	MYEOV, CCND1	African American	C	0.5751	1.0104	1.1513	1.22E-04
rs6504950	17q22	STXBP4	European ancestry	G	0.6551	1.0166	1.0743	0.0452
rs3745185	19p13	BABAM1	African American	G	0.7775	1.0119	1.1853	3.85E-05
rs2363956	19p13.11	ANKLE1	European ancestry	T	0.5136	1.0042	1.1365	1.92E-04
rs8170	19p13.11	BABAM1	European ancestry	A	0.1993	1.0066	1.1465	1.36E-03
ER+ breast cancer risk								
rs13387042	2q35	TNP1, DIRC3	European ancestry	A	0.7272	1.0028	1.1077	0.0287
rs2981579	10q26.13	FGFR2	European ancestry	A	0.6021	1.0196	1.1004	0.0224
rs3112572	16q12	LOC643714	African American	A	0.2151	0.9970	1.1447	6.45E-03
rs3745185	19p13	BABAM1	African American	G	0.7705	1.0119	1.1375	8.84E-03
ER–breast cancer risk								
rs8170	19p13.11	BABAM1	European ancestry	A	0.1943	0.9951	1.1866	8.38E-03
rs2363956	19p13.11	ANKLE1	European ancestry	T	0.5069	1.0014	1.1823	1.44E-03
rs4245739	1q32.1	MDM4	European ancestry	C	0.2405	1.0135	1.1490	0.0198
rs10069690	5p15.33	TERT	European ancestry	T	0.5972	1.0313	1.3217	2.47E-07
rs1432679	5q33.3	EBF1	European ancestry	C	0.7956	1.0240	1.2758	2.92E-04

a : risk allele frequency in AMBER imputation data.

b : the information metric from IMPUTE2.

c : the p-values were based on logistic regression between variant genotypes and breast cancer status while controlling for other covariates (see Methods).

Of the 74 GWAS-discovered variants, eight and fifteen have been reported to be associated with ER+ and ER- breast cancer, respectively [53–58] (Supplementary Table S2). We then tested the association of these particular variants with the corresponding breast cancer subtypes. Four of the eight GWAS-discovered ER+ breast cancer variants were found to affect ER+ breast cancer risk in the AMBER Consortium (nominal p-value < 0.05), including rs3112572 at chromosome 16q12, which was not replicated when tested for overall cancer risk (Table 1). For ER- breast cancer, five of the fifteen GWAS-discovered ER- breast cancer variants were associated, including rs4245739 at chromosome 1q32, which was not associated with overall breast cancer risk (Table 1). Taking into account these additional replicated loci by ER status, the replication rate increased to 27.0% (20/74) for all variants, and 71.4% (5/7) and 22.4% (15/67) for the variants discovered in populations of African and non-African ancestry, respectively. These observations are consistent with the notion that it is increasingly difficult to replicate GWAS findings across populations as the replication population becomes more genetically distant from the GWAS population.

### Dissecting the replicated GWAS signals

We next used the preferential LD approach to search for nearby (±500 kb) candidate causal variants or better markers with lower association p-values for the 20 GWAS-discovered variants replicated in the AMBER consortium. A total of 5,451 candidates were identified by our approach from 127,697 variants in the 1000 Genomes variant catalog that lie within 500 kb of the GWAS-discovered variants (0.29−0.85 candidates per 1 kb for each GWAS-discovered variant). Among these, 77,608 of the 1000 Genomes variants including 4,932 of the preferential LD selected candidates were successfully genotyped or imputed in the AMBER consortium and were tested for association with breast cancer risk. After accounting for the pairwise correlations among these variants, the effective number of independent tests was 19,617 [59]. Using a Bonferroni correction for the effective number of independent tests, we required a significance level of 2.55 × 10^−6^ to reach study-wide significance. We compared the candidate variants selected by our approach with all 1000 Genomes variants to evaluate the performance of the preferential LD approach for association with breast cancer risk.

**Figure 1:**
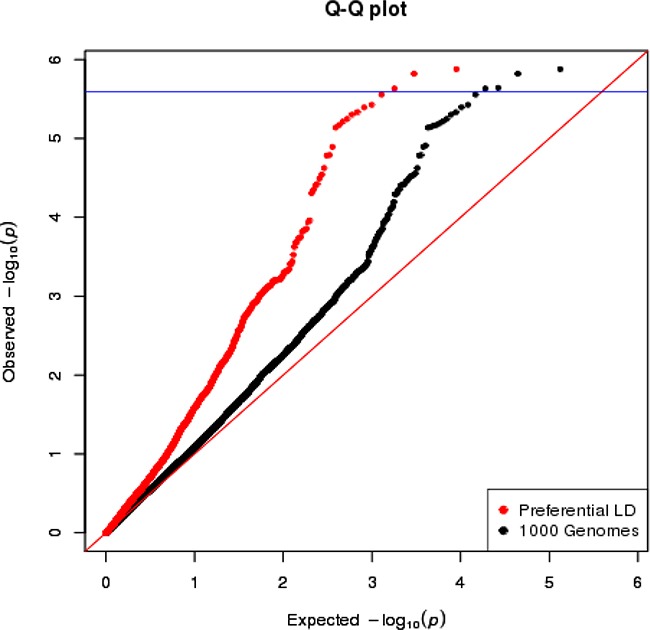
The QQ plot of overall breast cancer association p-values in AMBER consortium The variants selected by the preferential LD approach in the 18 replicated loci are in red. The 1000 Genomes variants in the same 18 loci are in black. The blue horizontal line corresponds to the study-wide significance cutoff 2.55×10^−6^.

**Figure 2:**
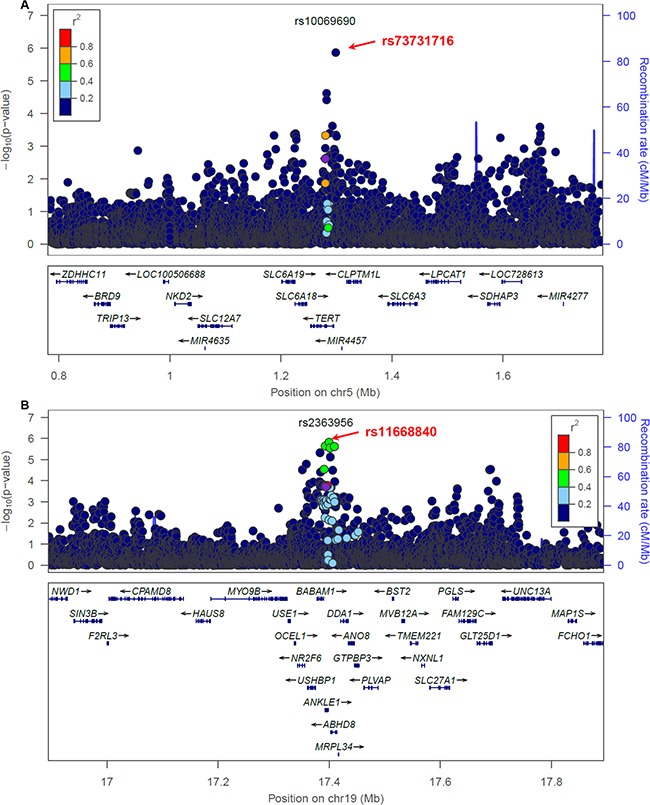
Breast cancer association of variants within 500 kb of rs10069690 **A.** and rs2363956 **B.** in the AMBER cohort. The GWAS-discovered variants were denoted by the purple circles.

We first focused on the 18 loci where the GWAS-discovered variants were associated with overall breast cancer risk in the AMBER consortium (Table 2). In general, we observed that markers with lower p-values are more enriched among the candidate variants selected by the preferential LD approach than among all neighboring 1000 Genomes variants (Figure 1). Four variants passed the study-wide significance cutoff: rs73731716 (p=1.33×10^−6^) in *TERT* locus and three variants in *ANKLE1-BABAM1* locus, rs11668840, rs8100241, and rs12982058 (p=1.51×10^−6^, 2.29×10^−6^, and 2.33×10^−6^ respectively) (Table 3 and Figure 2). All four variants except rs8100241 were selected by the preferential LD approach. In the ANKLE1-BABAM1 locus, variants rs8100241 and rs12982058 were no longer significant after conditioning on rs11668840 (p=0.7897 and 0.7954 respectively), suggesting the three variants represent the same signal. Previously, Chen et al found rs11668840 to be the strongest signal in this locus only for ER- breast cancer in AAs. For overall breast cancer risk in AAs, rs3745185, instead, was the most significant variant in this locus [53]. In the AMBER consortium, we found rs11668840 to be the most significant variant associated with both overall breast cancer and ER- breast cancer. For 14 of the 18 loci, the preferential LD approach was able to identify a better marker with a more significant p-value than the GWAS-discovered variant. Furthermore, for seven loci, the preferential LD approach was able to select the best marker with the lowest association p-value among all neighboring 1000 Genomes variants. In contrast, the best marker can only be found in one locus if selecting fine-mapping variants based on high LD with the GWAS signals (r^2^>0.6). For an additional six loci, the best candidate identified by the preferential LD approach was among the top 10 best markers in the corresponding locus. It is worth noting that for 11 of the above 13 loci, the best candidates identified by this approach were only in weak LD (r^2^<0.6) with the GWAS-discovered variants, which demonstrates the ability of this approach to pinpoint candidates even when they were not strongly linked with the GWAS-discovered variants. For example, rs73731716, the best marker identified by preferential LD approach by following up the GWAS signal rs10069690, is also the best marker among the 6,912 tested 1000 Genomes variants at the surrounding *TERT* locus. It is significantly associated with overall breast cancer risk with p-value 1.33×10^−6^ but only has weak LD with the GWAS signal (r^2^=0.015).

**Table 2:** The performance of the preferential LD approach in identifying the best markers in the GWAS loci^a^

GWAS-discovered Variants		Best marker in the 500kb neighborhood					Best marker among the preferential LD candidates					
rsID	p-value	rsID	Neighboring Genes	Imputation Quality	p-value	r^2^,^b^	rsID	Neighboring Genes	Imputation Quality	p-value	Rank^c^	r^2^
Overall breast cancer risk												
rs4849887	6.85E-03	rs4849899	LINC01101, GLI2	0.9967	5.64E-06	0.233	rs4849899	LINC01101, GLI2	0.9967	5.64E-06	1/5883	0.233
rs13000023	6.13E-04	rs185147777	DIRC3	0.8926	2.72E-04	0.012	rs113674867	LOC101928327, DIRC3-AS1	1.016	1.15E-03	10/6036	0.951
rs16857609	4.16E-03	rs185147777	DIRC3	0.8926	2.72E-04	0.001	rs78037304	DIRC3	1.001	6.13E-03	65/6178	0.054
rs13387042	0.0365	rs185147777	DIRC3	0.8926	2.72E-04	0.007	rs56269701	LOC101928327, DIRC3-AS1	1.0057	5.73E-04	2/6005	0.391
rs10069690	2.40E-03	rs73731716	TERT, MIR4457	0.9720	1.33E-06	0.015	rs73731716	TERT, MIR4457	0.972	1.33E-06	1/6912	0.015
rs1432679	2.58E-03	rs116197733	LOC101927697, EBF1	0.6969	6.38E-04	0.01	rs60172775	EBF1	0.9998	4.48E-03	28/4844	0.821
rs9693444	0.0339	rs77271190	DUSP4, LINC00589	1.0115	3.99E-05	0.094	rs77271190	DUSP4, LINC00589	1.0115	3.99E-05	1/5446	0.094
rs1011970	0.0473	rs3731213	CDKN2A	1.0043	7.61E-04	0.031	rs143070667	CDKN2B-AS1	0.9993	1.22E-03	2/6241	0.026
rs2981578	4.99E-06	rs2912778	FGFR2	1.0095	3.75E-06	0.922	rs143014944	FGFR2	0.9821	7.46E-04	16/6444	0.025
rs2981579	1.30E-03	rs2912778	FGFR2	1.0095	3.75E-06	0.16	rs2912778	FGFR2	1.0095	3.75E-06	1/6438	0.160
rs1219648	0.0324	rs2912778	FGFR2	1.0095	3.75E-06	0.035	rs2912778	FGFR2	1.0095	3.75E-06	1/6422	0.035
rs2981582	0.0438	rs2912778	FGFR2	1.0095	3.75E-06	0.044	rs2912778	FGFR2	1.0095	3.75E-06	1/6432	0.044
rs3817198	0.0472	rs57936908	KRTAP5-5, FAM99A	0.9825	8.47E-04	0.003	rs74047514	MRPL23, MRPL23-AS1	0.9841	1.07E-02	31/6671	0.041
rs609275	1.22E-04	rs115894455	ORAOV1	0.9798	3.52E-05	0.011	rs625625	LINC01488, CCND1	1.0059	4.48E-05	4/5751	0.350
rs6504950	0.0452	rs16955774	STXBP4, HLF	1.0032	1.64E-03	0.003	rs114380381	STXBP4	0.9739	2.61E-02	44/4580	0.089
rs3745185	3.85E-05	rs11668840	ANKLE1, ABHD8	1.0679	1.51E-06	0.155	rs62126227	BABAM1	1.0081	4.64E-06	5/5593	0.781
rs2363956	1.92E-04	rs11668840	ANKLE1, ABHD8	1.0679	1.51E-06	0.519	rs11668840	ANKLE1, ABHD8	1.0679	1.51E-06	1/5558	0.519
rs8170	1.36E-03	rs11668840	ANKLE1, ABHD8	1.0679	1.51E-06	0.086	rs62126227	BABAM1	1.0081	4.64E-06	5/5572	0.034
ER+ breast cancer risk												
rs13387042	0.0287	rs56269701	LOC101928327, DIRC3-AS1	1.0039	3.51E-05	0.391	rs56269701	LOC101928327, DIRC3-AS1	1.0039	3.51E-05	1/6005	0.391
rs2981579	0.0224	rs59100826	FGFR2, ATE1	0.9847	9.24E-06	0.001	rs2912778	FGFR2	1.0049	1.22E-05	2/6438	0.160
rs3112572	6.45E-03	rs1112135	CASC16	0.9996	7.75E-07	0.231	rs35850695	TOX3	0.9928	1.33E-05	11/5839	0.036
rs3745185	8.84E-03	rs10416082	PGLS	0.9055	7.96E-05	0.001	rs62126227	BABAM1	1.0092	1.11E-03	12/5593	0.781
ER- breast cancer risk												
rs8170	8.38E-03	rs11668840	ANKLE1, ABHD8	1.0658	1.49E-07	0.086	rs62126227	BABAM1	1.0068	2.94E-04	11/5572	0.034
rs2363956	1.44E-03	rs11668840	ANKLE1, ABHD8	1.0658	1.49E-07	0.519	rs11668840	ANKLE1, ABHD8	1.0658	1.49E-07	1/5558	0.519
rs4245739	0.0198	rs12405987	LINC00628, PPP1R15B	0.9511	4.33E-04	0.005	rs12064622	PLEKHA6	0.9951	1.65E-03	4/5109	0.028
rs10069690	2.47E-07	rs10069690	TERT	1.0313	2.47E-07	1	rs6867141	TERT	0.9451	1.31E-05	2/6912	0.067
rs1432679	2.92E-04	rs12332693	EBF1	1.0174	2.50E-04	0.919	rs60172775	EBF1	1.0043	5.92E-04	10/4844	0.821

a : the p-values were based on logistic regression between variant genotypes and breast cancer status while controlling for other covariates (see Methods).

b : calculated from the 1000 Genomes African population using Haploview.

c : the rank of the best marker identified by the preferential LD approach among all variants from the 1000 Genomes African population in the 500kb neighborhood of the GWAS-discovered variant.

**Table 3:** The 1000 Genome variants that passed study-wide significance when tested for association with breast cancer risk

rsID^a^	Position	Neighboring Genes	Allele	Frequency	Imputation Quality	OR	p-value^b^	Conditional p-value^c^
Overall breast cancer risk								
**rs73731716**	5:1298680	TERT, MIR4457	G	0.1073	0.9720	1.3123	1.33E-06	-
**rs11668840**	19:17399625	ANKLE1, ABHD8	C	0.4081	1.0679	0.8493	1.51E-06	-
rs8100241	19:17392894	ANKLE1	A	0.3980	1.0114	0.8476	2.29E-06	0.7897
**rs12982058**	19:17409380	ABHD8	T	0.3995	1.0130	0.8477	2.33E-06	0.7954
ER+ breast cancer risk								
rs1112135	16:52639755	CASC16	T	0.3250	0.9996	1.2420	7.75E-07	-
rs4238750	16:52639236	CASC16	T	0.3250	1.0003	1.2417	7.90E-07	-
ER–breast cancer risk								
**rs11668840**	19:17399625	ANKLE1, ABHD8	C	0.4132	1.0658	0.7589	1.49E-07	-
rs10069690	5:1279790	TERT	T	0.5972	1.0313	1.3217	2.47E-07	-
**rs61494113**	19:17401859	ANKLE1, ABHD8	A	0.4019	0.9992	1.3153	2.51E-07	0.1068
**rs12974508**	19:17401521	ANKLE1, ABHD8	T	0.3983	1.0045	0.7594	4.10E-07	0.9825
**rs12982058**	19:17409380	ABHD8	T	0.4044	1.0129	0.7614	4.23E-07	0.7954
rs8100241	19:17392894	ANKLE1	A	0.4032	1.0099	0.7610	4.28E-07	0.7897
**rs28473003**	19:17406167	ABHD8	T	0.3443	0.9850	1.3092	9.79E-07	0.1241

a : the variants selected by the preferential LD approach are in bold.

b : the p-values were based on logistic regression between variant genotypes and breast cancer status while controlling for other covariates (see Methods).

c : p-value after conditioning on rs11668840.

**Figure 3:**
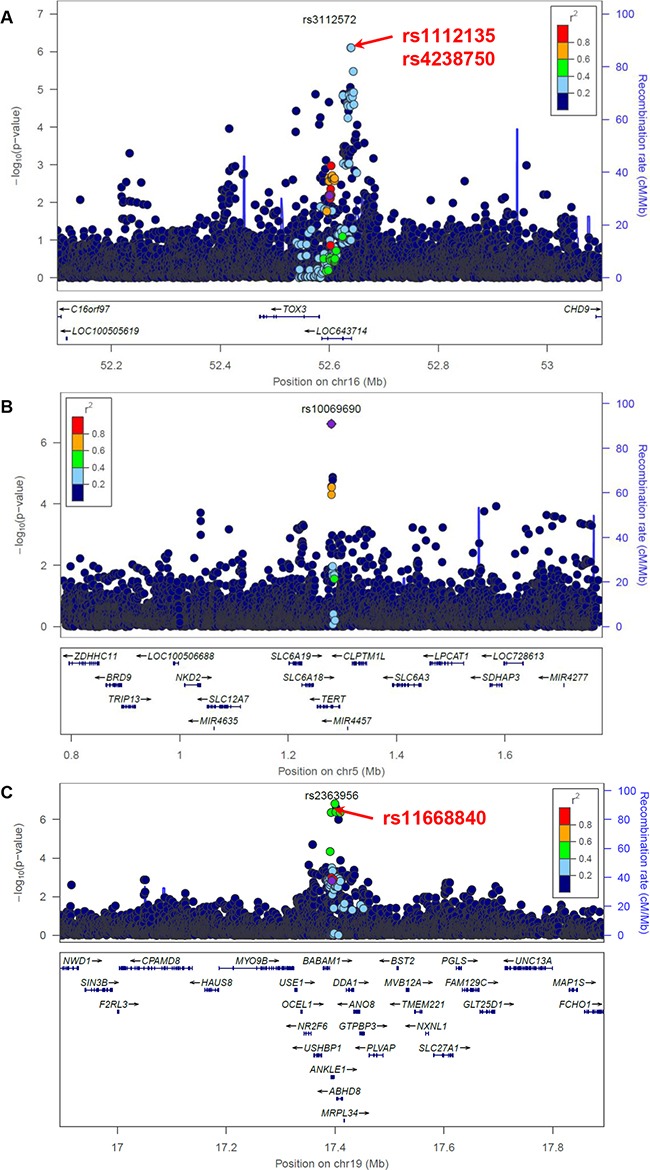
Association between variants within 500 kb of rs3112572 and ER+ breast cancer **A.**, between variants within 500 kb of rs10069690 **B.** and rs2363956 **C.** and ER- breast cancer in the AMBER cohort. The GWAS-discovered variants were denoted by the purple circles.

We next investigated the 9 loci where the GWAS-discovered variants were associated with breast cancer by ER status in the AMBER consortium (Table 2). The association between ER+ breast cancer and two variants in the neighborhood of GWAS-discovered variant rs3112572, rs1112135 and rs4238750, passed study-wide significance (Table 3 and Figure 3). These two variants were not included in the preferential LD candidates because they are more common than the GWAS-discovered variant rs3112572 in the 1000 Genomes African population (see Method). We will loosen up this requirement in future trans-ethnic applications of the preferential LD approach, as the allele frequencies can change substantially when the follow-up population is not the same as the GWAS population. In two replicated ER+ breast cancer loci, the best candidates identified by the preferential LD approach were among the top 10 best markers (Table 2). These include rs2912778 (p=1.22×10^−5^, ranked no.2 among 6438 tested 1000 Genomes variants) for the locus surrounding GWAS signal rs2981579 (p=0.0224), and rs56269701 (p=3.51×10^−5^, ranked no.1 among 6005 tested 1000 Genomes variants) for the locus surrounding GWAS signal rs13387042 (p=0.0287). The association between ER- breast cancer and seven 1000 Genomes variants passed study-wide significance, including the GWAS-discovered variant rs10069690 and six variants in *ANKLE1-BABAM1* locus (Table 3). All six variants except rs8100241 were selected by the preferential LD approach but none were significant after conditioning on rs11668840. In four of the five replicated ER- breast cancer loci, the best candidates identified by the preferential LD approach were among the top 10 best markers (Table 2).

### An example: dissecting a GWAS signal with known causal variant within FGFR2 locus in AA

**Figure 4:**
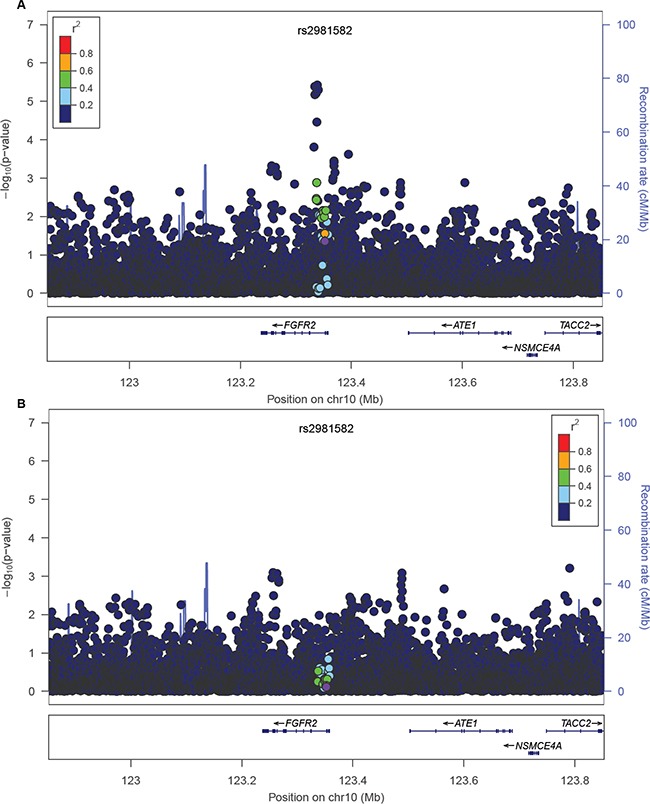
Breast cancer association of variants within 500 kb of rs2981582 in the AMBER cohort The GWAS-discovered variant rs2981582 is denoted by the purple circle. The -logP values before **A.** and after **B.** conditioning on the causal variant rs2981578 were shown.

FGFR2 locus was one of the first breast cancer loci identified by GWAS. The most strongly associated GWAS-variant is rs2981582, a non-coding variant in intron 2 of *FGFR2*, which encodes the fibroblast growth factor receptor 2 [7]. Further fine-mapping studies led to the identification of rs2981578 as the most likely causal variant in this locus [8, 60, 61]. The variant rs2981578 resides in a FoxA1 binding site in an enhancer of FGFR2 gene. The cancer risk allele (C) triggers stronger FoxA1 and PolII binding, enhanced transcription activity, and increased FGFR2 expression level [8, 60, 61]. The GWAS-discovered variant rs2981582, a tag of the causal variant, correlates with rs2981578 in European and Asian populations (r^2^=0.64 and 0.31 respectively), but the correlation is much lower in AAs (r^2^=0.05). Consistent with the LD, the association between rs2981582 and breast cancer risk is very strong in European individuals, more modest in Asian individuals, and mostly attenuated in AAs [8, 61]. In the AMBER Consortium, we also found rs2981582 was only nominally associated with overall breast cancer risk (p=0.0438). Nevertheless, rs2981578 was still the most likely causal variant of this locus in African-descent populations (p=4.99×10^−6^). After conditioning on the causal variant rs2981578, the major association signal in this locus was completely eliminated (Figure 4). The MAF for rs2981578 is much lower than the GWAS-discovered variant rs2981582 in the 1000 Genomes AFR population (7.9% and 48.8% respectively). When using the preferential LD approach to follow up rs2981582, we assumed HapMap II variants from YRI population (~2.9 M markers) as the genotyped variants in the original GWAS because the variants in the GWAS genotyping platform, an early SNP array at Perlegen Sciences with 266,732 variants, were not available (see Method). Although this assumption resulted in the removal of causal variant rs2981578 from the preferential LD candidates as it is a HapMAP variant in YRI population, our approach did identify rs2912778, which was most strongly associated with breast cancer in this locus in the AMBER consortium (p=3.75×10^−6^), as a candidate causal variant. Variant rs2912778 is also a non-coding variant in intron 2 of *FGFR2* and it is highly correlated with the causal variant rs2981578 (r^2^=0.92, MAF= 8.5% in 1000 Genomes AFR population). This finding further confirms the ability of the preferential LD approach to identify rarer and weakly tagged causal variants in a trans-ethnic setting.

## DISCUSSION

In this study, we investigated the associations of 74 breast cancer risk variants previously discovered by GWAS in 8,315 AA women from the AMBER consortium. We found that the majority of the GWAS-discovered variants identified from non-African populations could not be replicated in AA women in the AMBER consortium, which is consistent with the literature. Previously, Long *et al.* investigated 67 GWAS-discovered variants in 1,231 AA cases and 2,069 controls, and found that only 10 of them (14.9%) were significantly associated (p<0.05) with overall breast cancer risk and by subtype. Chen et al. examined 19 risk variants identified by GWAS and Feng et al. further tested an additional 54 GWAS-discovered variants in AA women [53, 62]. Together, they showed that 12 of the 73 GWAS-discovered variants (16.4%) could be replicated in their study population of 5,761 AA women at p<0.05. A recent large multi-consortium (including AMBER) fine-mapping study of breast cancer risk variants revealed that 10 of 73 (13.7%) tested GWAS hits were associated with breast cancer in 6,522 AA cases and 7,643 controls (under review, Haiman et al.). The consistently low replication rate from studies conducted by us and others [63, 64] reinforce the conclusion that it is challenging to extrapolate the GWAS variants identified from non-African populations to African ancestry populations, and highlight the challenge of trans-ethnic follow-up studies of GWAS hits.

Besides sample size and statistical power, a number of additional factors could contribute to the low replication rate and therefore have direct influence on the trans-ethnic application of the preferential LD approach. First, the causal variants can be population specific [4, 14]. For example, the causal variants of nondiabetic end-stage renal disease in *APOL1* gene are common in AAs but absent in European Americans [65]; the cardiomyopathy causal variant at *MYBPC3* is common in individuals from South Asia but not observed elsewhere [66]; and the *ABCA1* variant that reduces cholesterol efflux is Native-American ancestry specific [67]. As the GWAS-discovered variants, in general, are not the disease causing mutations but are linked to the causal variants, GWAS association signals resulting from a population-specific causal variant may disappear in other populations. Evidence is emerging that rare variants significantly contribute to cancer susceptibility including bladder cancer [23], ovarian cancer [19], glioma [27], prostate cancer [28, 29], and colorectal cancer [68–70]. As rare variants tend to be population specific, the cross-population replication rate of GWAS findings in cancer is expected to be low. Second, even if the causal variants are identical across populations, they may have different effect size and allele frequencies [14]. For a replication population where the effect size or allele frequency of the causal variant is lower than in the discovery population, a much larger sample size is required to identify the same signal. Third, the tagging efficiency of the variants in the genotyping platform differs in populations [4, 5]. As has been seen in the *FGFR2* locus, the GWAS-discovered variant in European population, rs2981582, is no longer a good proxy of the causal variant in AAs. With all of these considerations, well-powered breast cancer GWAS in African populations will not only improve the replication rate of the GWAS findings from other populations, but will also lead to identification of novel breast cancer loci that are specific to African ancestry. To date, there are only two moderately sized (~3,000 cases and ~3,000 controls) GWAS of breast cancer focused on women of African ancestry [40, 41], indicating the under representation of this population. In addition, as most commercially existing genotyping arrays were designed with a focus on populations of European descent, the overall genomic coverage based on LD is reduced in other populations, especially in populations of African ancestry, which are known for increased genome diversity and decreased levels of LD [5]. Therefore, the causal variants in African populations may not be well-captured using the existing genotyping platforms. Illumina has released its newly designed Infinium Multi-Ethnic Genotyping Array, which empowers GWAS studies in understudied populations including African-ancestry populations. Knowing that the GWAS signals exist in the populations of interest is essential to the success of follow-up studies using the preferential LD approach.

For the 20 loci where the GWAS-discovered variants were replicated in AA women in the AMBER consortium, we applied the preferential LD approach to search for causal variant candidates or better markers for AA breast cancer risk. The preferential LD approach could identify better markers in 16 of the 20 loci, and in 13 of them, the approach could identify markers that were top 10 among all genotyped and imputed 1000 genomes variants in the corresponding locus, which indicates the ability of this approach to follow up GWAS hits trans-ethnically. The preferential LD approach was developed to identify causal variants by following the GWAS-discovered variants even if the causal variants are much rarer. It is important to note that the goal of the preferential LD is to follow up a particular GWAS signal to search causal variants driving the corresponding association instead of fine mapping a GWAS locus (eg. selecting variants based on LD pruning) where additional risk variants independent of the GWAS signal can exist. The success of this approach relies on two factors: the presence of the causal variant in the variant catalog used by the approach, and the GWAS-discovered variant being the best tag for the causal variant. However, there were limitations for both of these two assumptions in the current trans-ethnic study. First, the currently best publicly available variant resource for African Population is from the 1000 Genomes Project, which includes 246 individuals of African-descent. As the causal variants of breast cancer are likely rare in the populations without breast cancer, they are expected to be depleted in this variant catalog, which was compiled from a relatively small number of healthy individuals. In our previous intra-ethnic application of the preferential LD approach, we utilized a comprehensive genome-wide variant catalog from 479 deeply sequenced individuals of European ancestry [15]. Being an understudied population, a comprehensive variant catalog is not readily available for populations of African descent. The recent availability of the extensive variant catalog from the UK10K project [71] and the Haplotype Reference Consortium [72] will significantly boost the genetic studies in European populations. The African Genome Variation Project (AGVP) [73] released recently and the Consortium on Asthma among African-ancestry Populations in the Americas (CAAPA) [74] are filling the gap for African populations. In contrast to CAAPA, the variants in AGVP were generated from genotyping and low-coverage sequencing and therefore rare variants may still be underrepresented in this dataset. Second, as described above, most of the GWAS-discovered variants in breast cancer were discovered in non-African population. Thus, the assumption of the GWAS-discovered variants being the best tags for the causal variants in African population is likely violated. Despite these limitations, we found that the preferential LD approach performed reasonably well in dissecting the GWAS signals in the AMBER consortium. Future studies with a more comprehensive variant catalog of African ancestry and the completion of well-powered breast cancer GWAS in African populations will be needed to better reveal the causal variants of breast cancer risk in AA women. In that case, the preferential LD approach can be directly carried out using the variant catalogs of AAs to follow up the AA GWAS-discovered variants, which is expected to be more powerful than the trans-ethnic application of this approach. On the other hand, given the promising trans-ethic performance of the preferential LD approach observed in this study, we anticipate more and more trans-ethic application of this approach in other human traits to be carried out in the future, especially when GWAS in the population of interest is not available.

## MATERIALS AND METHODS

### The AMBER consortium

The AMBER Consortium [32, 33] was formed in 2011 by combining data and biospecimens from four of the largest epidemiological studies of breast cancer in AA women: the Carolina Breast Cancer Study (CBCS) [42], the Women's Circle of Health Study (WCHS) [43, 44], the Black Women's Health Study (BWHS) [45] and the Multiethnic Cohort Study (MEC) [46].

The CBCS is a North Carolina population-based case control study of breast cancer. Breast cancer cases were identified using Rapid Case Ascertainment in cooperation with the NC Central Cancer Registry. Controls were identified using Division of Motor Vehicles lists for women under age 65 and Health Care Financing Administration lists for women 65 and older. The WCHS is a multi-site case control study in New York City (NYC) and New Jersey (NJ) aimed at evaluating risk factors for early and aggressive breast cancer in AA and European American (EA) women. Recruitment in NYC involved hospital-based ascertainment of cases, while controls were identified through random digit dialing (RDD). Cases in NJ were identified by the NJ State Cancer Registry using rapid case ascertainment. Controls were recruited though RDD and community-based efforts [47]. The BWHS is an ongoing prospective cohort study of health and illness among AA women, with a focus on cancer. Women diagnosed with breast cancer are identified by self-report in follow-up questionnaires, and confirmed by medical records, state cancer registries and the National Death Index. The MEC is a prospective cohort study that was designed to provide prospective data on cancer and other chronic diseases. Identification of incident breast cancer in study participants is by regular linkage with the Los Angeles County Cancer Surveillance Program and the State of California Cancer Registry. Controls in BWHS and MEC were chosen from among participants without breast cancer, and were frequency matched to cases on geographical region, sex, race, and 5-year age group.

All study participants provided consent for using their data and specimens for research purposes and the study was approved by Institutional Review Boards at participating institutions. ER status for cases was determined using pathology data from hospital records or cancer registry records.

### Preferential LD approach

Our approach identifies candidate causal variants from a comprehensive variant catalog with four major steps [15]. First, we select variants that are: 1) in a 1 Mb interval centered on a GWAS-discovered variant, 2) have not been evaluated in the GWAS of interest, and 3) are rarer than the GWAS-discovered variant. Second, we identify the candidate variants that are preferentially tagged by the GWAS-discovered variant by calculating the preferential LD statistic, which estimates the percentage of all GWAS investigated variants that can tag the candidate variant better than or as well as the GWAS-discovered variant. Third, we perform permutation tests and keep the candidate variants that have non-random LD with the GWAS-discovered variant. Finally, we prioritize the candidate variants that are preferentially tagged by the GWAS-discovered variant and are functionally important on the basis of a sorting score that incorporates both the preferential LD statistic and evolutionary conservation. Candidate variants with statistically significant sorting scores are considered to be the candidates for causal variants driving the association between the GWAS-discovered variant and the phenotype of interest.

We used the preferential LD approach to follow up the GWAS-discovered breast cancer risk variants that were replicated in the AMBER consortium by utilizing the variant catalog from the 1000 Genomes African population (phase I release 3). As the preferential LD approach excludes variants that were already interrogated in the corresponding GWAS from the search of candidate causal variants, we used the variants in the HapMap phase II YRI (Yoruba in Ibadan, Nigeria) samples as the variants analyzed for meta-GWAS, where multiple genotyping platforms and imputation were used, or when the GWAS platform content was unavailable (Supplementary Table S3). When assessing the performance of the preferential LD approach, we compared the candidate causal variants selected by our approach to all 1000 Genome variants that were in the 1Mb interval centered on the GWAS-discovered variants and were available in the AMBER dataset.

### Genotyping, quality control, and imputation

The GWAS-discovered variants and their corresponding followed-up variants selected by the preferential LD approach were added as part of the custom content to the Illumina Human Exome Beadchip v1.1 [48–51], and genotyped in CBCS, WCHS, and BWHS by the Center for Inherited Disease Research (CIDR). Variants successfully genotyped were subjected to stringent quality control (QC) metrics. Variants that are monomorphic variants, failed CIDR technical filters, or had missing rate ≥ 2%, > 1 Mendelian error in 17 HapMap trios, or > 2 discordant calls between 192 duplicate samples were omitted. DNA from a total of 6,828 unique participants from CBCS, WCHS, and BWHS were successfully genotyped and passed sample-level QC, including removal of samples with missing rate ≥ 2% and samples with unresolved identity. Variants from the 1000 Genomes project (Phase I v3, 11/23/2010) were imputed using SHAPEIT2 and IMPUTE2. In addition, 1528 AA subjects from MEC were previously genotyped with Illumina 1M-Duo chip and imputed with the 1000 Genomes reference panel. The genotyped and imputed genotypes for the CBCS, BWHS, and WCHS were combined with the MEC data to generate a complete AMBER analytical dataset. Variants were excluded if the minor allele frequency (MAF) was less than 0.6%, the imputation info score (INFO) was less than 0.5 in either set, or if the allele frequencies between the two sets differed by > 0.15.

### Statistical analysis

As principal component analysis (PCA) using variants genotyped in all four studies of the AMBER consortium revealed little heterogeneity across studies, we analyzed all studies jointly. PCA analysis identified 35 population outliers. These outliers and six additional samples with missing phenotype information were omitted from association analyses. We used PLINK [52] to test the association between allelic dosage and susceptibility to breast cancer, ER+ breast cancer, and ER- breast cancer using logistic regression. Study, age, geographic location, DNA source, and principal components from the PCA analysis that had p-value <0.1 in the covariate-only models for the corresponding phenotype were included as covariates in the association models.

## SUPPLEMENTARY TABLES


