# Supplementary material for: Trans-ethnic follow-up of breast cancer GWAS hits using the preferential linkage disequilibrium approach
Source: Oncotarget. 2016 Nov 4;7(50):83160–76. doi: 10.18632/oncotarget.13075 (PMC5341253; doi:10.18632/oncotarget.13075)
Supplement: Supplementary file 1 [file oncotarget-07-83160-s001.pdf]

Trans-ethnic follow-up of breast cancer GWAS hits using the preferential linkage disequilibrium approach

SUPPLEMENTARY TABLES

Supplementary Table S1: The GWAS-discovered variants evaluated in the AMBER consortium

GWAS-discovered Variants	First Author	PUBMEDID	GWAS Population
rs11249433, rs16886165, rs2981579, rs999737	Thomas G	19330030	European ancestry
rs13387042, rs3803662	Stacey SN	17529974	European ancestry
rs2180341	Gold B	18326623	Ashkenazi Jews
rs865686, rs4415084	Fletcher O	21263130	European ancestry
rs1219648	Hunter DJ	17529973	European ancestry
rs8170	Antoniou AC	20852631	European ancestry
rs10069690, rs1011970, rs10759243, rs10771399, rs10995190, rs11199914, rs11242675, rs11814448, rs11820646, rs12422552, rs12493607, rs1292011, rs132390, rs13281615, rs13329835, rs1432679, rs1436904, rs1550623, rs16857609, rs17356907, rs17817449, rs2016394, rs204247, rs2236007, rs2588809, rs2823093, rs3757318, rs3760982, rs3817198, rs3903072, rs4808801, rs4849887, rs4973768, rs527616, rs6001930, rs614367, rs616488, rs6472903, rs6504950, rs6762644, rs6828523, rs704010, rs7072776, rs720475, rs7904519, rs889312, rs941764, rs9693444, rs9790517	Michailidou K	23535729	European ancestry
rs11075995, rs12710696, rs4245739, rs6678914	Garcia-Closas M	23535733	European ancestry
rs2363956	Purrington KS	24325915	European ancestry
rs2380205	Turnbull C	20453838	European ancestry
rs2981582	Easton DF	17529967	European ancestry
rs2046210	Zheng W	19219042	Chinese
rs13000023, rs16886165, rs12355688, rs2981578, rs609275, rs3112572, rs3745185	Chen F	21852243	African American

Supplementary Table S2: The GWAS-discovered variants that associated with breast cancer subtypes

Breast cancer subtype	GWAS-discovered varaint
ER+	rs3112572, rs3745185, rs2981579, rs13387042, rs16886165, rs11249433, rs999737, rs3803662
ER−	rs10069690, rs1432679, rs2363956, rs8170, rs4245739, rs12710696, rs6678914, rs3757318, rs6001930, rs616488, rs11075995, rs10995190, rs17817449, rs10771399, rs17356907

Supplementary Table S3: The parameters used when running the preferential LD approach

GWAS-discovered Variants	Variant Catalog Population[a]	Genotyping Platform[b]	MAF Cutoff[c]	HWE Cutoff[d]
rs2981579	1KGafr	Illumina550v1	0.01	-
rs13387042	1KGafr	Illumina300v1	0.001	1E-10
rs1219648	1KGafr	Illumina550v1	0.01	-
rs8170	1KGafr	Illumina610Quad	0.05	1.0E-07
rs13000023, rs2981578, rs3112572, rs3745185, rs609275	1KGafr	Illumina1MDuo⋃HapMapIIr21YRI	0.01⋃0.05	-
rs10069690, rs1011970, rs1432679, rs16857609, rs3817198, rs4849887, rs6504950, rs9693444, rs4245739, rs2363956, rs2981582	1KGafr	HapMapIIr22YRI	0.01	1.0E-06

[a]: the variant catalog included single nucleotide variants (SNVs) from the 1000 Genomes phase I release 3 AFR population.
[b]: the genotyping platform used in the corresponding GWAS. SNVs from the HapMap phase II YRI samples were used when the GWAS is a meta-GWAS or when the GWAS platform content is unavailable. ⋃: union.
[c]: the MAF cutoff used to filter out variants in the corresponding GWAS. If a cutoff value is not given in the publication or the GWAS is a meta-GWAS, a default value 0.01 is used. ⋃ is used to connect the MAF cutoff used for each genotyping platform.
[d]: the p-value cutoff of Hardy–Weinberg Equilibrium (HWE) test used to filter out variants in the corresponding GWAS. If a cutoff value is not given in the publication or the GWAS is a meta-GWAS, a default value 1E-6 is used. -: variants were not filtered out based on HWE test in the corresponding GWAS.
